# Epigenetic Aging Mediates the Association of Diet Quality and Cognition in Late Adulthood

**DOI:** 10.1093/geroni/igaf122.2560

**Published:** 2025-12-31

**Authors:** Marrium Mansoor, Elayna Seago, Benjamin Katz

**Affiliations:** Virginia Tech, Blacksburg, Virginia, United States; Virginia Tech, Blacksburg, Virginia, United States; Virginia Tech, Blacksburg, Virginia, United States

## Abstract

Diet quality is a major factor that affects physical and cognitive health in later life. One possible mechanism for this may be through epigenetic modifications, including DNA methylation (DNAm). DNAm is associated with epigenetic age acceleration (EAA). EAA can be measured by epigenetic clocks that quantify the level of DNAm to provide an estimate of an individual’s biological age. GrimAge is an epigenetic clock that is associated with cardiovascular risk factors and predicts mortality and morbidity. Links between diet quality and EAA with cognition in later life have not yet been fully examined in a representative sample of older adults in the United States. We drew from 1792 participants (Mage = 70.3, 59.3% female, 78% white) from the Health and Retirement Study to examine whether EAA mediated the relationship between diet quality and cognitive performance in later life. Diet was assessed using adherence to the Dietary Approaches to Stop Hypertension (DASH) dietary pattern that optimize cardiovascular health by prioritizing consumption of vegetables, fruits, whole grains, fish, poultry, beans and nuts. Mediation models were used to examine whether EAA mediated the association between diet quality and performance on Serial 7s, Number Series, Immediate, and Delayed Recall, while controlling for age, gender, race, and years of education. We found that EAA fully mediated the relationship between diet and cognition for all four measures, with higher DASH scores leading to lower EAA and higher cognitive performance. These results reinforce the importance of diet quality on multiple aspects of later health, including cognition.

